# Supplementary material for: Axonal injury signaling is restrained by a spared synaptic branch
Source: eLife. 2025 Oct 29;13:RP104896. doi: 10.7554/eLife.104896 (PMC12571483; doi:10.7554/eLife.104896)
Supplement: Supplementary file 1. [file elife-104896-supp1.docx]

**Supplementary Table 1:** Summary of negative results from genetic manipulations that impair synaptic transmission and/or signaling at synapses. The listed UAS lines, UAS-RNAi lines and genetic mutations were tested for their ability to alter the expression of *puc*-lacZ in motoneurons. Outcomes and experimental details are noted.

| **Name**[LS1] | **Stock ID** | **Target/Function** | **Ref(s)** | **Driver (Gal4)** | **Observation(s)** |
| --- | --- | --- | --- | --- | --- |
| **UAS lines** |  |  |  |  |  |
| UAS-*shibire*-ts | RRID:BDSC_44222 | dominant temperature sensitive mutation in dynamin - confers rapid inhibition of synaptic vesicle endocytosis and exocytosis. | [(Kitamoto, 2001)](https://paperpile.com/c/4wrvWm/sBT9) | m12 | temperature shift to 32C for 24h. (nSyb-Gal4, UAS-Shi-ts flies are paralyzed when shifted to 32C for 20 minutes). No difference between shifted (32C) and unshifted (25C) for uninjured, single branch injured, and complete axotomy. (shift was done immediately after laser surgery). |
| UAS-dORK-  delta-C | RRID:BDSC_8928 | constitutively open K+ rectifying channel. Confers electrical silencing. | [(Nitabach et al., 2002)](https://paperpile.com/c/4wrvWm/xxFEt) | m12 | No difference from control (CS) flies, both uninjured and full axotomy. |
| UAS-dORK-  delta-NC (non-  conducting) | RRID:BDSC_6587 | Non-conducting version of UAS-ORK1 (control) | [(Nitabach et al., 2002)](https://paperpile.com/c/4wrvWm/xxFEt) | m12 | No difference from control (CS) flies, both uninjured and full axotomy. |
| UAS-gtACR1 | RRID:BDSC_92983 | light-inducible Cl- channel | [(Mohammad et al., 2017)](https://paperpile.com/c/4wrvWm/FPl1) | m12 | Kept in dark until 24h before dissection, when larvae were shifted to 530 nm light (for 24h). No difference between vehicle (ethanol) and ATR (1mM) control. As a control for the method, BG380Gal4; UAS-gtACR1 (ATR) flies showed rapid paralysis with shifted 530 nm light, but animals raised with vehicle food did not. |
| UAS-*Sh*-DN | RRID:BDSC_40974 | mutated Shaker K+ channel which attenuates membrane excitability | [(Mosca et al., 2005)](https://paperpile.com/c/4wrvWm/C7D8P) | m12 | No difference from control (CS) flies, uninjured |
| UAS-tkv-CA | RRID:BDSC_36537 | constitutively activated type I BMP receptor (thickveins) |  | BG380 | causes elevation of Gbb/BMP signaling (measured by pSMAD), but does not alter the induction of puc-lacZ in either injured (24h nerve crush) or uninjured animals. |
| **endogenous mutations** |  |  |  |  |  |

| *paralytic (para)-ts* |  | Temperature-sensitive allele of voltage-gated sodium channel | (Pittendrigh et al, 1997) |  | tested 6h at 37C, and 24h at 29. No difference between shifted and unshifted for uninjured animals. Also no difference between shifted and unshifted for full axotomy. (shift was done immediately after nerve crush). |
| --- | --- | --- | --- | --- | --- |
| Dm-*piezo* KO |  | *piezo* mechanosensitive Ca2+ channel | [(Kim et al., 2012)](https://paperpile.com/c/4wrvWm/YmUi0) |  | Similar to control for *puc*-lacZ induction |
|  |  |  |  |  |  |
| **RNAi lines** |  |  |  |  |  |
| *cacophony (cac*) | vdrc 101478 | alpha1 subunit of voltage-gated calcium channel |  | m12 with dicer2 | no difference from UAS-control (luciferase) RNAi |
|  |  |  |  | BG380, Dicer2 | no difference from UAS-control (luciferase) RNAi |
| *straightjacket (stj)* | RRID:BDSC_25807 | alpha 2 delta subunit of voltage-gated calcium channel |  | BG380, Dicer2 | no difference from UAS-control (luciferase) RNAi |
|  |  |  |  | D42 | no difference from UAS-control (luciferase) RNAi |
| *paralytic (para)* | RRID:BDSC_31471 | voltage-gated  sodium channel |  | BG380 | lethal |
| *paralytic (para*) | RRID:BDSC_31676 | voltage-gated  sodium channel |  | BG380, D42 | no difference from UAS-control (luciferase) RNAi |
| *shaker (sh)* | RRID:BDSC_31680 | shaker (sh) voltage gated K+ channel |  | BG380, Dicer 2 | no difference from UAS-control (luciferase) RNAi |
| *shaker cognate (shaw)* | RRID:BDSC_28346 | voltage gated K channel Kv3.1 |  | BG380, Dicer 2 | no difference from UAS-control (luciferase) RNAi |
| *synaptosomal-associated protein (SNAP25)* | RRID:BDSC_27306 | t-SNARE, regulation of neurotransmitter release |  | BG380, Dicer2 | no difference from UAS-control (luciferase) RNAi |
|  | RRID:BDSC_34377 |  |  | BG380, Dicer2 | no difference from UAS-control (luciferase) RNAi |
|  |  |  |  | D42 | no difference from UAS-control (luciferase) RNAi |
| *synaptobrevin (nSyb)* | RRID:BDSC_31983 | v-SNARE, role in synaptic vesicle exocytosis |  | BG380, Dicer2 | no difference from UAS-control (luciferase) RNAi |
|  |  |  |  | m12 | no difference from UAS-control (luciferase) RNAi |
| *syntaxin 4 (syx4)* | RRID:BDSC_44054 | tSNARE, role in synaptic vesicle exocytosis |  | BG380, Dicer2 | no difference from UAS-control (luciferase) RNAi |
| *syntaxin 1A (syx1A)* | RRID:BDSC_25811 | tSNARE, role in synaptic vesicle exocytosis |  | BG380, Dicer2 | no difference from UAS-control (luciferase) RNAi |
| *synaptotagmin 4 (syt4)* | RRID:BDSC_39016 | calcium dependent exocytosis of secretory vesicles. |  | BG380, Dicer2 | no difference from UAS-control (luciferase) RNAi |
| *vesicular glutamate transporter (vGlut)* | v104324 | vesicular glutamate transporter |  | BG380, Dicer2 | no difference from UAS-control (luciferase) RNAi |
|  | RRID:BDSC_40927 |  |  | D42 | no difference from UAS-control (luciferase) RNAi |
|  |  |  |  | BG380, Dicer2 | no difference from UAS-control (luciferase) RNAi |
| *piezo* | vdrc 2796 | mechanosensitive Ca2+ channel |  |  | no difference from UAS-control (luciferase) RNAi |
| *no mechanoreceptor potential C (nompC)* | vdrc 105579 | pore-forming subunit of mechanotransduction channel |  | BG380, Dicer2 | no difference from UAS-control (luciferase) RNAi |
|  |  |  |  |  |  |
| *shortwing (sw)* | vdrc 101559 | intermediate chain subunit of dynein |  | BG380, Dicer2 | no difference from UAS-control (luciferase) RNAi |
|  | vdrc 48334 |  |  | BG380, Dicer2 | no difference from UAS-control (luciferase) RNAi |
| *neurexin 1*  *(nrx-1)* | vdrc 36326 | presynaptic protein, cell-cell interactions, exocytosis, synaptic signaling |  | BG380, Dicer2 | no difference from UAS-control (luciferase) RNAi |
|  | RRID:BDSC_27502 |  |  | BG380, Dicer2 | no difference from UAS-control (luciferase) RNAi |
|  |  |  |  | D42 | no difference from UAS-control (luciferase) RNAi |
|  | RRID:BDSC_32408 |  |  | BG380, Dicer2 | no difference from UAS-control (luciferase) RNAi |
|  |  |  |  | D42 | no difference from UAS-control (luciferase) RNAi |
| *neuroligin 1 (nlg1)* | vdrc 42616 | postsynaptic cell surface protein, cell adhesion, excitatory synapses |  | BG380, Dicer2 | no difference from UAS-control (luciferase) RNAi |
| *defective proboscis extension response 4 (dpr4)* | vdrc 28519 | cell surface receptor for DIPs, adhesion protein, synapse organization |  | BG380, Dicer2 | no difference from UAS-control (luciferase) RNAi |
|  | vdrc 102905 |  |  | BG380, Dicer2 | no difference from UAS-control (luciferase) RNAi |
|  | vdrc 28518 |  |  | BG380, Dicer2 | no difference from UAS-control (luciferase) RNAi |
| *fasciculin II (fasII)* | RRID:BDSC_34084 | cell adhesion molecule, axonal pathfinding/guidance |  | BG380, Dicer2 | no difference from UAS-control (luciferase) RNAi |
|  | vdrc 8393 |  |  | BG380, Dicer2 | no difference from UAS-control (luciferase) RNAi |
|  | vdrc 36350 |  |  | BG380, Dicer2 | no difference from UAS-control (luciferase) RNAi |
| *comatose/NSF1 (comt)* | RRID:BDSC_27890 | N-ethylmaleimide-sensitive factor 1, role disassembly of SNARE complexes |  | D42 | no difference from UAS-control (luciferase) RNAi |
| *semaphorin 2b (sema2b)* | RRID:BDSC_28932 | secreted semaphorin, involed in embryonic axonal guidance |  | BG380, Dicer2 | no difference from UAS-control (luciferase) RNAi |
| *plexin B (plexB)* | RRID:BDSC_57813 | Acts as a receptor for Sema-2a and seems to suppress axon branching |  | BG380, Dicer2 | no difference from UAS-control (luciferase) RNAi |
| *neuroglian (Nrg)* | RRID:BDSC_37496 | L1-type cell adhesion molecule |  | BG380, Dicer2 | no difference from UAS-control (luciferase) RNAi |
| *wishful thinking (wit)* | RRID:BDSC_25949 | BMP type II receptor with known functions synaptic development and maintenance at the Drosophila NMJ |  | BG380, Dicer2 | no effect on *puc*-lacZ induction in uninjured animals. *puc*-lacZ induction 24 post peripheral nerve crush is similar to wild type animals. |
|  | vdrc 103808 |  |  | BG380, Dicer2 | " |
| *thickveins (tkv)* | RRID:BDSC_41904 | BMP type I receptor with known functions synaptic development and maintenance at the Drosophila NMJ |  | BG380, Dicer2 | no effect on *puc*-lacZ induction in uninjured animals. *puc*-lacZ induction 24 post peripheral nerve crush is similar to wild type animals. |
